# Structural Reversibility of Optic-Disc Cupping in Glaucoma: Pathophysiology, Imaging Assessment, and Clinical Implications

**DOI:** 10.3390/jcm14248897

**Published:** 2025-12-16

**Authors:** Gloria Roberti, Carmela Carnevale, Manuele Michelessi, Lucia Tanga, Sara Giammaria, Francesco Oddone

**Affiliations:** IRCCS-Fondazione Bietti, 00198 Rome, Italy; gloria.roberti@fondazionebietti.it (G.R.);

**Keywords:** glaucoma, optic disc cupping, intraocular pressure, medical treatment, surgical treatment, optic nerve head photography, visual field, confocal scanning laser ophthalmoscopy, optical coherence tomography

## Abstract

**Background/Objectives**: Reversibility of glaucomatous optic-disc cupping, following intraocular pressure (IOP) reduction, represents a fascinating structural response observed in both pediatric and adult patients. This review summarizes evidence on its mechanisms, diagnostic evaluation, and clinical significance. **Methods**: A comprehensive review of experimental, clinical, and imaging-based studies investigating optic-disc cupping reversibility was conducted. Findings were categorized by patient population, imaging technique, and follow-up duration. **Results**: Experimental models established a strong correlation between IOP reduction and optic-disc structural recovery. Pediatric glaucoma demonstrated the greatest reversibility due to enhanced ocular tissue elasticity, whereas adult cases showed limited yet measurable structural changes after sustained IOP lowering. Imaging modalities, including confocal scanning laser ophthalmoscopy and spectral-domain optical coherence tomography (SD-OCT), consistently confirmed quantitative disc-shape changes correlated with pressure reduction. **Conclusions**: Although optic-disc cupping reversal reflects biomechanical and glial remodeling rather than true neuronal recovery, it remains an important biomarker of successful IOP control. Advanced imaging provides valuable insights into optic-nerve-head (ONH) biomechanics and may improve glaucoma management.

## 1. Introduction

Reversibility of glaucomatous cupping refers to the improvement in optic-disc configuration following therapeutic intraocular pressure (IOP) lowering, a phenomenon first described in 1869 [1]. Subsequent research has confirmed its occurrence in experimental animal models, as well as in pediatric and adult glaucoma patients. The reversibility of cupping has been assessed with various imaging modalities, from early fundus photography and stereoscopic analysis to modern spectral-domain optical coherence tomography (SD-OCT), now considered the gold standard. Thus, the reversibility of optic-nerve-head cupping was expressed differently across studies: as a percentage change, as a volumetric measurement in cubic microns, as a measurement of neural-tissue area, or as a thickness measurement expressed in microns.

It is good to note that a rapid postoperative IOP drop may induce transient optic-disc edema (‘pseudoreversal’), which typically resolves within two months [2].

This review aims to summarize key evidence on the mechanisms, imaging findings, and clinical implications of optic-disc cupping reversibility.

We conducted a PubMed search using the terms “optic-disc cupping reversal,” “glaucoma,” “IOP reduction,” and “structural reversal,”. The search included studies published between 1982 and 2025. We included human or experimental studies reporting structural optic-nerve-head changes after intraocular pressure reduction, with no restrictions on age, glaucoma subtype, or imaging modality. Exclusion criteria were: non-English language, reviews or editorials, non-glaucomatous cupping etiologies, and studies lacking structural outcome measures.

## 2. Experimental Studies

Experimental investigations have played a fundamental role in elucidating the mechanisms underlying optic-disc cupping reversal following IOP reduction. Among the most influential contributions are the studies conducted by Shirakashi and colleagues, who established a quantitative link between IOP reduction and morphologic changes in the optic nerve head (ONH) in primate models of glaucoma [3,4].

Shirakashi et al. demonstrated a significant correlation between the percentage of IOP reduction and the percentage of reversal in multiple optic-disc parameters in adult monkeys. In their model, one eye of each monkey underwent repeated argon laser photocoagulation of the trabecular meshwork to induce chronic ocular hypertension (OHT). Stereoscopic optic-disc photographs were obtained before treatment and every one to two weeks thereafter using a simultaneous stereoscopic fundus camera (Topcon TRC-SS, Topcon Corp., Tokyo, Japan). The slides were digitized and analyzed with a computerized image analysis system (Topcon IMAGEnet, Tokyo, Japan), which calculated several topographic parameters, including the vertical and horizontal cup-to-disc ratios (CDR[V], CDR[H]), neuroretinal rim area (RA), rim area-to-disc area ratio (RA/DA), and cup volume (CV).

For each animal, the maximum reversal—defined as the greatest reduction in CDR(V), CDR(H), and CV/DA or increase in RA/DA—was determined, and the percentage of maximum reversal was calculated. Transient cupping reversal was observed during spontaneous IOP reduction in all eyes. The mean duration of maximum reversal was 4.9 ± 2.0 weeks, associated with a mean IOP decrease of 34.3 ± 19.3%. The mean maximum percentage reversals in CDR(V), CDR(H), RA/DA, and CV/DA were 11.8 ± 7.0%, 12.5 ± 6.7%, 30.6 ± 12.1%, and 37.0 ± 14.4%, respectively. A statistically significant correlation was observed between the magnitude of IOP reduction and the degree of reversal for each parameter. The authors proposed that anterior displacement of the cup base due to decompression might contribute to the apparent improvement in disc configuration, although irreversible glaucomatous damage remained evident in nearly all eyes [3].

In a subsequent 12-month study, Shirakashi et al. further analyzed the dynamics of cupping reversal in five monkey eyes with experimental glaucoma. Spontaneous IOP reduction was observed throughout follow-up and divided into two stages: stage 1 (first four months, representing early glaucoma) and stage 2 (final four months, corresponding to more advanced disease). The authors compared the extent of cupping reversal associated with equivalent IOP reductions between the two stages. During stage 1, significant decreases were noted in both vertical and horizontal CDRs and in the CV/DA ratio, along with a significant increase in the RA/DA ratio. Although similar directional changes were observed during stage 2, the magnitude of reversal was significantly smaller for any given level of IOP reduction. The authors concluded that while structural improvement of the optic disc occurs across all stages of glaucoma, the potential for reversal diminishes as damage progresses, likely due to reduced residual tissue elasticity and volume. This finding implies that the biomechanical capacity for ONH recovery following pressure reduction decreases in advanced disease stages [4].

## 3. Reversibility of Optic-Disc Cupping in Pediatric Glaucoma Patients

### 3.1. Pathophysiology

**Figure 1:**
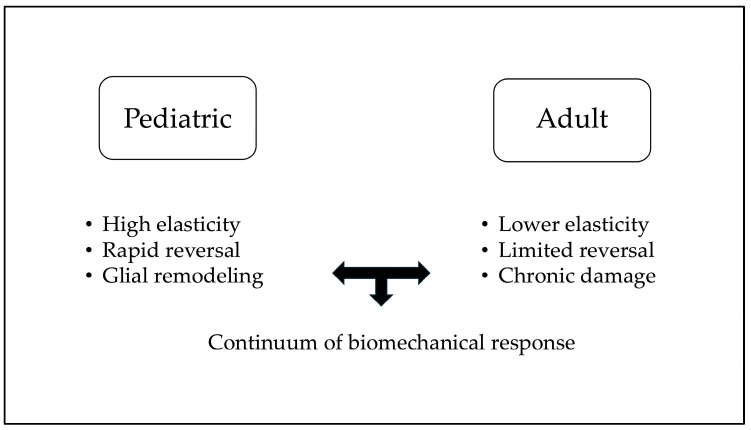
Pediatric vs. Adult structural reversal of disc cupping.

Reversal of glaucomatous optic-disc cupping has been extensively documented in pediatric glaucoma, particularly after successful surgical reduction of IOP. Early reports indicated that optic-disc cupping reversal occurs in approximately 40–50% of childhood glaucoma cases following prompt IOP control [5,6,7,8]. The phenomenon is far more frequent and pronounced in infants and young children compared to adults, reflecting developmental differences in ocular tissue biomechanics. A schematic flowchart summarizing pathways of structural reversibility in pediatric versus adult eyes is reported in Figure 1.

Several hypotheses have been proposed to explain the mechanisms underlying this reversibility. Shaffer (1969) [5] first suggested that improvement in optic-disc morphology might result from astroglial regeneration after ischemic stress is relieved. Elevated IOP primarily affects astroglial cells before significant ganglion cell death occurs; following IOP normalization, these glial elements may proliferate and partially fill the optic cup [9].

Alternative theories emphasize the mechanical and structural properties of the infant eye. Disc excavation in congenital glaucoma likely arises from posterior displacement of the lamina cribrosa and expansion of the scleral canal under elevated IOP [10]. Because ocular tissues in infancy are more elastic and collagen-deficient, this mechanical deformation is more readily reversible once pressure is lowered. Elastic recoil of the lamina and scleral canal may restore preexisting geometry, reducing cupping and increasing the RA [11,12].

However, normalization of cup size is not universal. When IOP elevation is prolonged or severe, irreversible loss of ganglion cells and axons may preclude meaningful structural recovery [10]. Therefore, the timing of IOP normalization remains a critical determinant of both anatomical and functional reversibility.

### 3.2. Diagnosis

Assessment of optic-disc morphology in children remains challenging due to limited cooperation, nystagmus, and media opacities (e.g., corneal edema secondary to elevated IOP).

Historically, several studies defined surgical success solely by IOP reduction without evaluating ONH or functional outcomes.

Meirelles et al. (2008) evaluated changes in CDR[V] in 45 eyes of 36 children with glaucoma after surgery and observed a mean reduction in CDR from 0.75 ± 0.15 to 0.62 ± 0.22, paralleling a significant IOP decrease [13]. Multivariate analysis revealed that younger age and earlier surgical intervention were predictive of greater reversal, confirming that the first 6–12 months of life represent a critical therapeutic window [14,15].

Reversal typically occurs within weeks after IOP reduction. Kessing and Gregersen (1977) reported resolution of glaucomatous cupping within two weeks to three months in 12 of 18 eyes with congenital glaucoma postoperatively, while Quigley (1982) observed optic disc improvement in 40% of eyes within six weeks after successful surgery [6,14].

Objective quantification of ONH changes was initially achieved with fundus photography and later refined with OCT. Schwartz et al. (1985) first used computerized stereophotogrammetry to document decreased cupping and visual field (VF) improvement following IOP reduction in both pediatric and adult patients [16]. However, fundus photography has inherent limitations: image acquisition is difficult in infants, and delineation of disc margins may be unreliable due to poor focus, nystagmus, or corneal haze [17].

Mochizuki et al. (2011) [18] employed RetCam digital imaging to quantify ONH size changes after glaucoma surgery and found that decreases in disc area correlated with IOP and axial length reduction. They attributed cupping reversal to scleral canal shrinkage, suggesting that persistent canal enlargement indicates ongoing stress on the optic nerve. Nevertheless, the authors acknowledged that photograph-based analysis may be affected by manual delineation errors and variable image quality [18].

Overall, OCT-based morphometric evaluation now represents the gold standard for pediatric ONH assessment due to its reproducibility, quantitative precision, and ability to measure peripapillary retinal nerve fiber layer (RNFL) thickness in addition to disc morphology [17,18].

#### Correlation Between Cupping Reversal and RNFL Changes

The relationship between optic-disc cupping reversal and RNFL thickness remains controversial in pediatric glaucoma. Several studies have reported discordance between apparent structural reversal and OCT-derived RNFL measurements, suggesting that cupping improvement may not always reflect neuronal recovery [7,8,19,20].

Chang and Grajewski (2016) [19] described a 5-year-old girl who underwent trabeculectomy for primary glaucoma: despite visible cupping reversal and increased rim area, OCT revealed RNFL thinning postoperatively. The authors hypothesized that elevated preoperative IOP causes axoplasmic flow blockade, leading to transient axonal swelling and RNFL thickening; after IOP normalization, restored flow produces apparent thinning, reflecting decongestion rather than loss [19].

Similarly, Go et al. (2020) [20] reported a 10-month-old infant with primary congenital glaucoma in whom cup size decreased from 0.6 to 0.4 after goniotomy, with no change in RNFL thickness or Bruch’s membrane opening (BMO) [20]. Ely et al. (2014) [7] observed postoperative cupping reversal in 58% (19/33) of eyes with juvenile open-angle or congenital glaucoma but found stable or declining RNFL thickness in most cases [7].

Elhusseiny and VanderVeen (2021) [21] reported consistent findings in four pediatric cases, noting no significant RNFL changes despite IOP reduction [21]. Finally, Glaser et al. (2022) [8], studying very young children (mean age 1.14 ± 0.93 years) under anesthesia with SD-OCT, found frequent clinical cupping reversal after surgery without corresponding RNFL or macular structural improvement [8].

Collectively, these studies demonstrate that optic disc appearance alone is insufficient to assess true structural recovery in pediatric glaucoma. OCT-derived RNFL and macular parameters provide more reliable and objective indicators of disease status and prognosis.

It is important to highlight that the available literature under represents the most difficult-to-image children (for example, those with marked cornea edema or sever nystagmus), which could bias estimates of reliability and treatment effect. Therefore, future systematic reviews reporting the number of excluded/unusable images per study, whether OCT reliability indices or manual re-segmentation were used, and considering sensitivity analyses that account for image-failure bias are recommended.

Studies included in this review are summarized in Table 1.

## 4. Reversibility of Optic-Disc Cupping in Adult Glaucoma Patients

### 4.1. Fundus and Stereophotographic Imaging

Reports of optic-disc cupping reversal in adult patients first emerged in the 1980s. Pederson and Herschler (1982) [22] described six glaucoma patients who exhibited cupping reversal on sequential fundus photographs. Five patients underwent glaucoma filtering surgery, and one patient demonstrated rapid reversal following IOP normalization during a glaucomatocyclitic crisis. Complete filling of the cup was observed in one eye, while the others showed partial inward displacement of the cup walls and modest shallowing of the excavation. In one case, a subsequent IOP increase 10 months post-surgery resulted in renewed cupping progression. VF improvement was noted in only one patient. Mean IOP reduction averaged 27.5 mmHg, and the authors attributed the limited degree of reversal compared with pediatric cases to reduced scleral and laminar elasticity in adults [22].

Katz et al. (1989) [23] identified reversible cupping in 16 of 75 glaucomatous eyes using serial fundus photographs. Reversal occurred only in eyes achieving ≥30% IOP reduction; eyes with <20% reduction showed no improvement. Among eyes with ≥30% IOP lowering, 18 VFs demonstrated significant improvement (*p* < 0.05), supporting a correlation between structural and functional recovery [23].

Five-year follow-up data from the Collaborative Initial Glaucoma Treatment Study (CIGTS) confirmed that IOP reduction is associated with partial cupping reversal, though without consistent functional improvement [24]. In 348 eyes with baseline and five-year stereoscopic photographs, 66 eyes exhibited detectable disc configuration changes, of which 23 eyes (51%) showed measurable reduction in cup size. Surgical therapy was associated with a higher proportion of cupping reversal than medical therapy. Mechanistic hypotheses for these structural changes include redistribution of axoplasmic fluid, posterior re-entry of displaced fluid, relaxation of the lamina cribrosa, and glial proliferation [24].

### 4.2. Rodenstock Optic Nerve Head Analyzer

The first prospective study investigating cupping reversal associated with sustained IOP reduction in adults utilized the Rodenstock Optic Nerve Head Analyzer (G. Rodenstock Instrumente GmbH, Munich, Germany), providing objective quantification of optic disc parameters [25]. Shin et al. (1989) [25] included 13 patients with a sustained IOP reduction > 5 mmHg for at least two weeks. IOP was lowered via medical therapy alone in nine eyes, a combination of medical therapy and laser trabeculoplasty in two eyes, and trabeculectomy in two eyes. Mean IOP reduction was 48.3 ± 15.7%. Following treatment, mean CDR decreased significantly, and RA increased. The degree of reversal was strongly correlated with the percentage of IOP reduction, while the duration of pressure reduction (mean 13.6 ± 10.4 weeks) was not a significant factor [25].

Similarly, Tsai et al. (1991) [26] retrospectively analyzed 28 eyes from 28 patients and found increased RA and decreased CDR after IOP reduction. Eyes achieving ≥40% IOP lowering showed statistically significant improvement in global VF indices, whereas those with <35% reduction did not [26]. Rath et al. (1996) [27] further confirmed that both the magnitude of IOP reduction and the final mean IOP significantly influenced the extent of cupping reversal. Eyes exhibiting measurable cupping improvement had greater mean IOP reductions and lower post-treatment IOP than eyes with progressive deterioration, highlighting the role of aggressive and sustained pressure lowering in promoting ONH structural recovery [27].

### 4.3. Confocal Scanning Laser Ophthalmoscopy

Confocal scanning laser ophthalmoscopy, particularly using the Heidelberg Retina Tomograph (HRT; Heidelberg Engineering, Heidelberg, Germany), allows reproducible and objective measurement of optic-disc topography. Several studies have applied HRT to assess cupping reversal after IOP reduction in adult glaucoma patients.

Irak et al. (1996) [28] evaluated 49 patients before and at least three months after trabeculectomy to minimize peripapillary swelling from postoperative hypotony. Mean IOP decreased from 23.1 ± 6.8 mmHg to 12.7 ± 7.1 mmHg, corresponding to a mean reduction of 43.8% ± 29.9%. Thirty-four patients (69%) achieved >30% IOP reduction. Mean cup area, CV, and CDR decreased significantly from 1096 ± 572 µm^2^ to 960 ± 557 µm^2^, 335 ± 302 µm^3^ to 271 ± 245 µm^3^, and 0.51 ± 0.20 to 0.44 ± 0.20, respectively (*p* < 0.004). Rim area and rim volume increased significantly from 1009 ± 418 µm^2^ to 1141 ± 406 µm^2^ and 224 ± 160 µm^3^ to 258 ± 153 µm^3^, respectively. Changes in cup area, rim area, CDR, retinal cross-sectional area, and height in contour correlated significantly with both mean and percent IOP reduction. Cup and rim volumes were associated only with percent IOP change. Patients achieving ≥30% IOP reduction demonstrated greater reversal of cupping [28].

Park et al. (1997) [29] observed similar findings in 6 of 13 patients with at least 25% IOP reduction two months after trabeculectomy. Mean RNFL thickness increased from 0.135 ± 0.129 mm to 0.162 ± 0.091 mm, without reaching statistical significance. Segmental rim area analysis revealed the greatest increase in superotemporal and inferotemporal quadrants, suggesting that partial restoration of depressed neuronal tissue, in addition to improved blood flow and axoplasmic flow, may contribute to cupping reversal. VF changes were not assessed [29].

Lesk et al. (1999) [30] studied 20 glaucoma patients undergoing filtration surgery or tube shunt implantation, with VF assessment before and after surgery. Patients were divided into two groups based on IOP reduction: Group A (11–22%) and Group B (≥47%). In Group A, most HRT parameters worsened, whereas in Group B, most parameters improved. Changes in CDR, cup area, RA, rim and CV, and mean and maximum cup depth correlated significantly with percent IOP reduction. RNFL thickness and cross-sectional area were positively correlated with IOP reduction but did not reach statistical significance. VF improvement was observed in 1 of 3 patients in Group A (33%) and 5 of 13 patients in Group B (38%), with greater HRT-detected CDR improvement in patients with improved fields. Postoperative imaging was performed on average 6.2 months after surgery to avoid confounding by disc edema [30].

Topouzis et al. (1999) [31] enrolled 25 eyes in a prospective study, obtaining HRT images at 1–3 weeks, 3–6 months, and >6 months post-trabeculectomy. Initial postoperative imaging showed decreased CV and mean cup depth, with increased height variation contour, reflecting early disc improvement. No statistically significant changes were noted in cup area, rim volume, RA, CDR, or maximum cup depth. Subsequent imaging sessions showed that most topographic parameters returned to preoperative values, suggesting that early improvement may not persist in eyes with more advanced glaucoma [31].

Kotecha et al. (2001) [32] demonstrated long-term improvement in ONH appearance following IOP reduction in patients with early to moderate glaucoma, most evident after two years. RA increased globally, with segmental improvement localized to nasal regions with the least pre-existing damage. IOP reduction correlated significantly with changes in RA and cup depth, while age and baseline disease severity had no effect [32].

Tan et al. (2004) [33] retrospectively analyzed patients with OHT and primary open angle glaucoma (POAG) maintaining ≥25% IOP reduction for one year. Approximately one-third of eyes exhibited cupping reversal, predominantly in the nasal half, with about 20% showing sustained reversal for at least one year. The study emphasized using a customized experimental reference plane to minimize measurement artifacts due to IOP variation [33].

Figus et al. (2011) [34] conducted a six-month prospective study in 56 eyes post-trabeculectomy. Mean RNFL thickness increased significantly at 3 and 6 months postoperatively, whereas no significant changes were observed in ONH parameters, including RA, rim volume, maximum cup depth, cup area, CV or CDR, nor in VF indices [34].

Harju et al. (2008) [35] prospectively followed 51 patients with exfoliation glaucoma and 5 with OHT over 6 years. CV, mean cup depth, and maximum cup depth decreased significantly at 6 months and 2 years postoperatively, and changes persisted up to 6 years. Presence of cup reversal was associated with lower risk of glaucoma progression, likely due to reduced lamina cribrosa strain and decreased mechanical stress on retinal ganglion cell axons [35].

### 4.4. Spectral Domain Optical Coherence Tomography

SD-OCT has become the preferred imaging modality to quantitatively assess ONH morphology and structural reversibility after IOP reduction, providing high-resolution measurements of BMO, rim parameters, and peripapillary RNFL thickness.

Waisbourd et al. (2016) [36] conducted a prospective cohort study in 61 glaucoma patients divided into three groups based on baseline IOP: Group 1 (>32 mmHg), Group 2 (22–31 mmHg), and Group 3 (<22 mmHg, control). Groups 1 and 2 underwent IOP-lowering interventions, while Group 3 remained untreated. SD-OCT, automated perimetry, and transient visual evoked potentials (VEPs) were performed at three follow-up visits. Post-interventional structural improvements were most evident in Group 2, with some improvement also observed in Group 1. No significant changes were detected in controls. Average RNFL thickness remained unchanged in all groups. Functional reversibility was scarce; VF indices (mean deviation and pattern standard deviation) and VEP parameters did not significantly improve. Structural improvements were independent of the magnitude of IOP reduction, as both low- and high-pressure groups showed similar changes [36].

Díez-Álvarez et al. (2017) [37] analyzed corneal biomechanics via the Ocular Response Analyzer (ORA) and ONH morphology using SD-OCT before and three months after deep sclerectomy in 49 POAG patients. Postoperatively, mean IOP decreased by 27.9%, corneal resistance factor (CRF) decreased by 10.1%, and corneal hysteresis (CH) increased by 18.4%. SD-OCT revealed decreased mean cup depth and thickening of prelaminar tissue, with no significant changes in lamina cribrosa depth or thickness. The authors identified CRF as a primary preoperative factor influencing ONH structural changes, suggesting that higher CRF confers greater resistance to chronic IOP stress, permitting more pronounced tissue decompression postoperatively [37].

Longitudinal SD-OCT assessments following trabeculectomy have further elucidated structural reversal of disc cupping (SRDC). Gietzelt et al. (2018) examined 88 eyes undergoing trabeculectomy with mitomycin C at 3, 6, 12, and 18 months [38]. Kiessling et al. (2019) retrospectively analyzed 65 eyes undergoing ab interno trabeculectomy with or without combined phacoemulsification, grouped into three postoperative clusters: 1–6 months, 7–12 months, and 13–24 months [39]. In both studies, SRDC correlated with significant increases in BMO-minimum rim width (BMO-MRW), particularly within the first 12 months. Increases were less pronounced and of shorter duration following ab interno trabeculectomy. Morphometric improvements correlated with IOP reduction, while BMO-minimum rim area (BMO-MRA), RNFL thickness, and VF parameters remained largely unchanged [38,39].

Gietzelt et al. (2020) [40] analyzed SRDC following glaucoma drainage device implantation (Baerveldt and Ahmed valves) in 43 eyes of 39 patients. BMO-MRW and BMO-MRA increased significantly between baseline and early postoperative visits (20–180 days) in parallel with IOP reduction. However, midterm (181–360 days) and long-term (>360 days) follow-up revealed no statistically significant changes. RNFL thickness and VF indices remained stable [40].

Lüke et al. (2025) [41] evaluated SRDC after PRESERFLO^®^ MicroShunt (Santen, Osaka, Japan) implantation in patients with preoperative and follow-up SD-OCT at 3, 6, 12, and 18 months. Global BMO-MRW increased significantly within the first three months, correlating with IOP reduction. Peripapillary RNFL thickness remained unchanged at three months but decreased at later time points. Compared with conventional filtration surgery, SRDC following PRESERFLO^®^ was less pronounced and less persistent, likely due to partial early postoperative shunt occlusion and higher early IOP levels [41].

A limitation of many SD-OCT studies has been their retrospective design, necessitating clustering of follow-up visits due to irregular data collection [38,39,40]. To address this, Gietzelt et al. (2022) [42] conducted a prospective study on early postoperative SRDC after trabeculectomy. Twenty-four eyes were examined preoperatively and at day 1, days 2–3, and week 1. BMO-MRW increased significantly as early as day 1, persisting through day 3 and week 1, directly correlating with IOP reduction. RNFL thickness showed a small but significant increase at week 1. These results suggest that acute IOP reduction, rather than surgery per se, drives rapid morphometric changes in the ONH [42].

In summary, SD-OCT studies consistently demonstrate SRDC and increases in BMO-based parameters following surgical IOP reduction. Proposed mechanisms include relief of preoperative axonal compression and transient axonal edema, though the exact processes remain incompletely understood [38,40,42].

The evidence across studies is markedly heterogeneous, with substantial variability in patient populations, imaging platforms, reference planes, and analytic methods. This methodological diversity limits the comparability of reported structural outcomes and the ability to draw uniform quantitative conclusions.

An overview of studies involving adult patients with optic-disc cupping reversal is summarized in Table 2.

## 5. Discussion

Structural reversibility of optic-disc cupping represents a complex phenomenon influenced by the interplay between IOP reduction, ONH biomechanics, and residual neural tissue. Both pediatric and adult glaucoma patients demonstrate morphometric improvements following therapeutic interventions, although the magnitude, duration, and functional implications of these changes differ markedly between age groups and glaucoma severity.

It is important to note that most available studies on optic-disc cupping reversibility are limited by small sample sizes, retrospective designs, and heterogeneity in imaging modalities and measurement approaches, with inconsistencies in reference planes and differences in definitions of “reversal”, which should be considered when interpreting their findings.

Table 3 summarizes limitations and strengths of the imaging modalities reported.

Another source of heterogeneity lies in the wide range of follow-up intervals, spanning from immediate postoperative assessments to multi-year observations. Short-term evaluations mainly capture acute biomechanical decompression after IOP reduction, whereas longer-term follow-up may reflect a combination of tissue remodeling, disease progression, and measurement variability. Consequently, differences in timing reduce the comparability of structural reversibility outcomes across studies.

In pediatric glaucoma, ONH reversibility is facilitated by greater tissue elasticity and deformability. Studies report that 40–50% of eyes show optic-disc cupping reduction following early surgical IOP lowering [6,7,8]. The underlying mechanisms likely include astroglial regeneration, relief of tissue compression, and elastic recoil of the lamina cribrosa and scleral canal [9,10,12]. Despite clinical evidence of cupping reversal, corresponding peripapillary RNFL or macular retinal layer changes are often absent, suggesting that preoperative neural tissue loss may be irreversible even when disc appearance improves [7,8,20]. These findings emphasize that structural recovery of the optic disc does not necessarily equate to neural recovery in pediatric glaucoma.

In adult glaucoma, structural reversal is generally less pronounced, likely due to reduced scleral and laminar elasticity [22,23]. Substantial IOP reduction (>30–40%) appears necessary to induce measurable ONH morphometric changes, with surgical interventions achieving greater reversibility than medical therapy [24]. Proposed mechanisms include redistribution of axoplasmic flow, transient prelaminar axonal edema, glial proliferation, and biomechanical restoration of laminar and peripapillary tissue [24,25,26].

The prelaminar neural tissue thickening is based on several etiologies: shift of axonal fluid from the peripapillary RNFL; shift of fluid pushed through the lamina at higher IOP level and then redistributed posteriorly into the prelaminar axons after IOP reduction; some increase in axoplasmic transport blockade within the lamina cribrosa by the relative constriction of the laminar pores [23].

Despite these changes, peripapillary RNFL thickness and VF parameters often remain unchanged, highlighting the limited functional impact of cupping reversal in adults [36,38,39].

We may propose a unified conceptual framework in which SRDC is understood as a multifactorial response driven by (i) acute biomechanical decompression following IOP reduction, (ii) the elastic properties of the lamina cribrosa and scleral canal, (iii) redistribution of axoplasmic flow, and (iv) age-dependent glial and connective-tissue remodeling. Within this integrated perspective, pediatric and adult SRDC represent points along a continuum of biomechanical adaptability shaped by age, tissue stiffness, and disease chronicity, rather than two separate and unrelated phenomena.

Confocal scanning laser ophthalmoscopy studies corroborate the SD-OCT findings, showing early post-interventional reduction in cup area and volume, accompanied by rim thickening. However, the persistence of these changes varies with glaucoma stage, surgical technique, and magnitude of IOP reduction [28,31,32]. Similarly, HRT and fundus photographic analyses indicate that structural reversibility is more evident in early glaucoma and pediatric cases, whereas advanced disease or lower residual elasticity limits the degree of morphological recovery [33,34,35].

SD-OCT has refined our understanding of SRDC by enabling precise quantification of BMO-based parameters, rim width, and RNFL thickness over time. Early postoperative studies show that BMO-MRW increases within days after trabeculectomy and correlates closely with IOP reduction [42]. These findings suggest that acute mechanical decompression, rather than direct surgical manipulation, drives early structural changes. Longitudinal analyses indicate that while BMO-MRW and BMO-MRA may partially regress over months, early morphometric improvement serves as a sensitive biomarker for biomechanical response of the ONH [40,41].

Several critical points emerge from the current literature. First, structural improvement does not always translate into functional recovery, particularly in adults. Second, the degree of reversibility is strongly age- and stage-dependent, with pediatric and early-stage glaucoma showing the greatest morphometric changes. Third, biomechanical properties of the eye, such as CH and CRF, influence the extent of ONH recovery, highlighting the role of tissue compliance in structural reversibility [37]. Finally, early and sustained IOP reduction is consistently associated with maximal structural benefit, emphasizing the importance of timely intervention in both pediatric and adult populations.

Overall, SRDC should be interpreted as a marker of biomechanical and morphometric response rather than direct neural restoration. Clinical decisions should integrate structural imaging with functional assessment, acknowledging that apparent improvement in ONH appearance may not fully reflect preserved or recovered retinal ganglion cell function.

## 6. Conclusions

Structural reversibility of optic-disc cupping represents a significant, yet complex, aspect of glaucoma management. Evidence from both pediatric and adult populations demonstrates that therapeutic IOP reduction can induce measurable morphometric improvements in the ONH, particularly in the prelaminar region and in BMO-based parameters. Pediatric eyes, due to higher tissue elasticity and residual neural capacity, exhibit the most pronounced and rapid cupping reversal, whereas adult eyes generally show more modest and transient changes.

Despite structural improvements, functional recovery—as measured by peripapillary RNFL thickness, macular retinal layers, or VF indices—is often limited, especially in adults. This dissociation suggests that observed cupping reversal predominantly reflects biomechanical decompression, axoplasmic fluid redistribution, and glial remodeling rather than true restoration of lost retinal ganglion cells.

Several factors influence the degree and durability of reversibility, including patient age, stage of glaucoma, preoperative neural tissue integrity, magnitude and timing of IOP reduction, and ocular biomechanical properties such as CH and CRF. Surgical interventions that achieve substantial and sustained IOP lowering are associated with the greatest morphometric benefit, with early postoperative OCT-based measurements serving as sensitive indicators of ONH biomechanical response.

The methodological limitations of included studies underscore the need for future studies. Emerging priorities include: (i) prospective longitudinal studies with standardized OCT and BMO-based parameters; (ii) improved modeling of optic nerve head biomechanics to clarify the relative contributions of laminar deformation, scleral stiffness, and axoplasmic flow; (iii) harmonization of structural metrics across imaging platforms. Unresolved controversies—such as the dissociation between apparent cupping reversal and stable or declining RNFL thickness, and the clinical significance of early postoperative changes—require further investigation.

In clinical practice, structural reversal of cupping should not be regarded as a direct indicator of functional recovery, but rather as a complementary sign of effective IOP control. Even when morphological improvement is observed, it represents only one component of the overall evaluation and cannot substitute for a thorough assessment of visual function. A proper interpretation of cupping reversal therefore requires pairing high-resolution structural imaging—such as SD-OCT—with comprehensive functional testing, including standard automated perimetry, retinal sensitivity analysis, and, when appropriate, electrophysiological measures. Only the systematic integration of both structural and functional data enables reliable prognostication, precise monitoring of glaucomatous progression, and the tailoring of truly individualized therapeutic strategies in both pediatric and adult glaucoma patients.

## Figures and Tables

**Table 1:** Summary of clinical studies evaluating the reversal of glaucomatous cupping in pediatric patients.

Authors	Year	Study Population	Study Design	Follow-Up	Structural Assessment	Functional Assessment	Intervention	Key Findings
Cockburn D.M. [11]	1983	9-year-old-girl	Case report	4 months	Photographs	VF ^1^	Medical therapy	Photographs show reversal of the cupping over the follow-up period during which IOP ^2^ were brought into the normal range
Schwartz B. et al. [16]	1985	4 patients	Case report	NA ^3^	Stereophotographs	VF ^1^	Medical and surgical therapy	The decrease in IOP ^2^ was associated with a regression of visual field loss and a decrease in optic-disc cupping
Mochizuki H. et al. [18]	2011	29 patients	Retrospective	NA ^3^	RetCam digital ONH ^4^ photographs	NA ^3^	Surgery	In case of successful surgery, percent change in disc area is correlated to percent change in IOP ^2^ and axial length. The cupping reversal was also associated with a profound shrinkage of the scleral canal.
Ely A.L. et al. [7]	2014	28 patients	Retrospective	NA ^3^	Disc photography OCT ^5^	VF ^1^	Surgery	Cupping reversal was identified in 58% of patients after surgery. A prevalently stable average peripapillary RNFL ^6^ on OCT ^5^ was found whereas a minority showed continued RNFL ^6^ loss independently to the changes or not in optic disc cup
Chang T.C. et al. [19]	2016	5-year-old girl	Case report	3 months	Disc photography OCT ^5^	NA ^3^	Trabeculotomy	The successful IOP ^2^ reduction was associated with thinning of the RNFL ^6^ on OCT ^5^ despite a postoperative decrease of cupping and increase in neuroretinal rim area at clinical examination
Go M.S. et al. [20]	2020	10-month-old infant	Case report	NA ^3^	OCT ^5^	NA ^3^	Goniotomy	The decrease of the optic disc cup was not associated with a significant changes of the Bruch membrane opening size and peripapillary RNFL ^6^
Elhusseiny A.M. et al. [21]	2021	4 patients	Case report	From 3 weeks to 8 months	OCT ^5^	NA ^3^	Medical and surgical therapy	No significant changes in mean global peripapillary RNFL ^6^ after IOP ^2^ reduction
Glaser T.S. et al. [8]	2022	14 patients	Retrospective	From 35 to 595 days	Intraoperative OCT ^5^	NA ^3^	Surgery	The clinically observed ONH ^4^ cupping reversal after surgery was not associated with a corresponding change in global or sectoral peripapillary RNFL^6^ and in the 3 mm macular retinal volume or retinal-layer volumes

^1^ VF = visual field; ^2^ IOP = intraocular pressure; ^3^ NA = not applicable; ^4^ ONH = optic nerve head; ^5^ OCT = optical coherence tomography, ^6^ RNFL = retinal nerve fiber layer.

**Table 2:** Summary of clinical studies evaluating the reversal of glaucomatous cupping in adult patients.

Authors	Year	Study Population	Study Design	Follow-Up	Structural Assessment	Functional Assessment	Intervention	Key Findings
Pederson et al. [22]	1982	6 patients	Case series	NA ^1^	Fundus photography	VF ^2^	Glaucoma filtering surgery	The degree of anatomical reversal was less pronounced than that typically seen in infantile glaucoma
Katz et al. [23]	1989	55 patients	Retrospective	NA ^1^	Fundus photography	VF ^2^	Treatment not specified	Reversal occurred only in eyes achieving at least a 30% IOP ^3^ reduction. 18 visual fields were judged to have improved significantly
Parrish et al. [24]	2009	348 eyes	Prospective	5 years	Fundus photography	VF ^2^	Medical and surgical treatment	66 eyes exhibited detectable changes in disc configuration, visual field outcomes did not differ significantly
Shin et al. [25]	1989	13 POAG ^4^ patients	Prospective	13 weeks	RONA ^5^	NA ^1^	Medical, laser and surgical treatment	Decrease in mean cup-to-disc ratio and a corresponding increase in neuroretinal rim area following IOP ^3^ reduction. The duration of pressure reduction was not a significant factor
Tsai et al. [26]	1991	28 POAG ^4^ patients	Retrospective	NA ^1^	RONA ^5^	VF ^2^	Treatment not specified	Patients achieving ≥40% IOP ^3^ reduction demonstrated statistically significant improvement in global VF ^2^ indices, whereas those with <35% reduction did not.
Rath et al. [27]	1996	78 POAG ^4^ patients	Retrospective	NA ^1^	RONA ^5^	NA ^1^	Treatment not specified	27 of 78 eyes demonstrated a measurable decrease in optic-disc cupping showing significantly greater mean IOP ^3^ reductions compared to those with progressive disc deterioration
Irak et al. [28]	1996	42 POAG ^4^ patients, 2 PXG ^6^ patients, 2 PG ^7^ patients, 1 PACG ^8^ patient, 2 secondary glaucoma associated with inflammation.	Prospective	3 months	HRT ^9^	NA ^1^	Trabeculectomy	Significant postoperative decreases were observed in cup area, cup volume, and cup-to-disc area ratio, while rim area and rim volume increased significantly. These changes were strongly correlated with both mean and percent IOP ^3^ reductions.
Park et al. [29]	1997	13 POAG ^4^ patients	Prospective	2 months	HRT ^9^	NA ^1^	Trabeculectomy	Six of thirteen patients with at least a 25% IOP ^3^ reduction revealed that rim area increased most markedly in the superotemporal and inferotemporal regions. RNFL ^10^ thickness increased slightly.
Lesk et al. [30]	1999	21 POAG ^4^ patients	Prospective	26 weeks	HRT ^9^	VF ^2^	Tube shunt implantation or filtration surgery	Changes in cup-to-disc ratio, cup area, rim area, rim and cup volume, and mean cup depth were significantly correlated with percent IOP ^3^ reductions. Patients with improved visual fields showed greater HRT ^9^-detected improvement in cup-to-disc ratio.
Topouzis et al. [31]	1999	25 POAG ^4^ patients	Prospective case series	Beyond 6 months	HRT ^9^	NA ^1^	Trabeculectomy	Changes in the optic disc that may be present 2 weeks after a trabeculectomy do not appear to persist 4 and 8 months later in eyes with advanced glaucomatous optic nerve damage.
Kotecha et al. [32]	2001	95 POAG ^4^ patients	Prospective	2 years	HRT ^9^	NA ^1^	Trabeculectomy	Rim area increased progressively over time, particularly in the nasal region, Age and baseline disease severity did not influence the degree of reversal.
Tan et al. [33]	2004	40 subjects, 10 with OHT ^11^, 10 with POAG ^4^, and 20 normal controls	Retrospective	NA ^1^	HRT ^9^	NA ^1^	Medical treatment	Approximately one-third of eyes exhibited cupping reversal, predominantly in the nasal half, with about 20% showing sustained reversal for at least one year.
Harju et al. [35]	2008	51 PXG ^6^ patients and 5 OHT ^11^ patients	Prospective	6 years	HRT ^9^	VF ^2^	Trabeculectomy, laser and medical treatment	Presence of cup reversal was associated with lower risk of glaucoma progression.
Figus M. et al. [34]	2011	56 POAG ^4^ patients	Prospective	6 months	HRT ^9^	VF ^2^	Trabeculectomy	Mean RNFL ^10^ thickness significantly increased whereas no changes in functional parameters (MD ^12^ and PSD ^13^) and rim indices (area and volume) were detected
Waisbourd M. et al. [36]	2016	61 patients with any form of glaucoma	Prospective	From 3 to 12 months	SD-OCT ^14^	VF ^2^ VEP ^15^	Medication, laser, surgery, paracentesis, pulled Latina suture or a combination thereof	The significant and sustained reduction of IOP^3^ was associated with a significant structural improvement (cup-to-disc ratio and cup volumes) following intervention. No significant improvements in VEP ^15^, VF ^2^ MD ^12^ and PSD ^13^ parameters.
Díez-Álvarez L. et al. [37]	2017	49 POAG ^4^ patients	Prospective	3 months	SD-OCT ^14^	NA ^1^	Deep sclerectomy	Significant changes in both corneal and ONH ^16^ biomechanical properties were observed 3 months after surgery.
Gietzelt C. et al. [38]	2018	88 patients	Retrospective	From 3 to 18 months	SD-OCT ^14^	VF ^2^	Trabeculectomy with MMC ^17^	A significant increase in global and sectorial BMO-MRW ^18^ and BMO-MRA ^19^ measurements in eyes up to more than one year after surgery was detected. RNFL ^10^ thickness and VF ^2^ function seemed not to be affected.
Kiessling D. et al. [39]	2019	65 patients	Retrospective	From 1 to 24 months	SD-OCT ^14^	VF ^2^	Ab-interno trabeculectomy	The structural change was correlated to a significant increase in the BMO-MRW ^18^ especially at 1–6 months after surgery. VF ^2^ function did not change markedly
Gietzelt C. et al. [40]	2020	39 patients	Retrospective	From 1 to 18 months	SD-OCT ^14^	VF ^2^	Baerveldt and Ahmed valve devices	BMO-MRW ^18^ and BMO-MRA ^19^ significantly increase between baseline and short-term follow-up examinations 20 to 180 days after surgery. RNFL ^10^ and VF ^2^ showed no improvement
Gietzelt C. et al. [42]	2022	24 patients	Prospective	1 week	SD-OCT ^14^	NA ^1^	Trabeculectomy	The BMO-MRW ^18^ parameter strongly increased during the whole first week after surgery
Luke JN et al. [41]	2025	59 patients	Retrospective	From 3 to 18 months	SD-OCT ^14^	NA ^1^	PRESERFLO^®^ microshunt	BMO-MRW ^18^ increased significantly in the first 3 months after surgery. Peripapillary RNFL ^10^ thickness was unchanged after three months and significantly decreased in later follow-up.

^1^ NA = not applicable; ^2^ VF = visual field; ^3^ IOP = intraocular pressure; ^4^ POAG = primary open angle glaucoma; ^5^ RONA = Rodenstock Optic Nerve Head Analyzer; ^6^ PXG = pseudoexfoliative glaucoma; ^7^ PG = pigmentary glaucoma; ^8^ PACG = primary angle-closure glaucoma; ^9^ HRT = Heidelberg Retina Tomograph; ^10^ RNFL = retinal nerve fiber layer; ^11^ OHT = Ocular Hypertension; ^12^ MD = mean deviation; ^13^ PSD = pattern standard deviation; ^14^ SD-OCT = spectral domain optical coherence tomography; ^15^ VEP = visual evoked potentials; ^16^ ONH = optic nerve head; ^17^ MMC = Mitomycin C; ^18^ BMO-MRW = Bruch’s membrane opening minimum rim width; ^19^ BMO-MRA = Bruch’s membrane opening minimum rim area.

**Table 3:** Strengths and limitations of imaging modalities.

Modality	Strengths	Limitations	Key Parameters
Fundus Photography	Widely available	Low reproducibility	CDR ^1^
RONA ^2^	ONH ^3^ objective quantification	Not widely used	CDR ^1^, RA ^4^
HRT ^5^	3D topography	Reference plane errors	Cup volume
SD-OCT ^6^	High resolution	Segmentation issues	RNFL ^7^ thickness, BMO-MRW ^8^, BMO-MRA ^9^

^1^ CDR = cup disc ratio; ^2^ RONA = Rodenstock Optic Nerve Head Analyzer; ^3^ ONH = optic nerve head; ^4^ RA = rim area; ^5^ HRT = Heidelberg Retina Tomograph; ^6^ SD-OCT = spectral domain optical coherence tomography; ^7^ RNFL = retinal nerve fiber layer; ^8^ BMO-MRW = Bruch’s membrane opening minimum rim width; ^9^ BMO-MRA = Bruch’s membrane opening minimum rim area.

## Data Availability

No new data were created or analyzed in this study. Data sharing is not applicable to this article.

## Abbreviations

The following abbreviations are used in this manuscript:

BMO	Bruch’s membrane opening
BMO-MRA	BMO-minimum rim area
BMO-MRW	BMO-minimum rim width
CDR[V]	vertical cup-to-disc ratio
CDR[H]	horizontal cup-to-disc ratio
CH	corneal hysteresis
CRF	corneal resistance factor
CV	cup volume
HRT	Heidelberg retina tomograph
IOP	intraocular pressure
OHT	ocular hypertension
ONH	optic nerve head
ORA	ocular response analyzer
POAG	primary open angle glaucoma
RA	neuroretinal rim area
RA/DA	rim area-to-disc area ratio
RNFL	retinal nerve fiber layer
SD-OCT	spectral domain optical coherence tomography
SRDC	structural reversal of disc cupping
VEP	visual evoked potentials
VF	visual field
